# Assessment of graph literacy among German medical students– an observational cross-sectional survey study

**DOI:** 10.1186/s12909-025-07206-7

**Published:** 2025-04-25

**Authors:** Janina Soler Wenglein, Andreas Heidenreich, Hendrik Friederichs

**Affiliations:** 1 Medical Education Research Group, Medical School OWL, Bielefeld University, Bielefeld, Germany; 2 Department of Pediatrics, Medical School and University Medical Center OWL, Protestant Hospital of the Bethel Foundation, Bielefeld University, Bielefeld, Germany; 3 Laboratory of Experimental Pediatric Pneumology and Allergology, Center for Biomedical Education and Science (ZBAF), Department of Human Medicine, Faculty of Medicine, Witten/Herdecke University, Witten, Germany; 4 Institute of Social Medicine and Epidemiology, University of Luebeck, Luebeck, Germany

**Keywords:** Graph literacy, Information literacy, Semiotic activity, Undergraduate medical students

## Abstract

**Background:**

Information literacy depends on diverse skills in processing information, including understanding graphs properly. Especially for those advising and informing people with less information, health and graph literacy, it is important to achieve high competence in these areas themselves. Graph literacy, therefore, is a form of semiotic activity that is a crucial component of overall literacy for (future) physicians. We analyzed the graph literacy of undergraduate medical students to gain knowledge about their skills and potential areas for improvement.

**Methods:**

An observational cross-sectional survey study was performed with undergraduate medical students in their academic years 1 to 5 using the “Graph Literacy Scale.” It measures the participant’s ability to read and interpret graphically provided information with 13 questions in three dimensions regarding visual data: “reading the data,” “reading between the data,” and “reading beyond the data.” Participants can score between 0 and 13 points.

**Results:**

We obtained 449 complete questionnaires. Undergraduate medical students showed above-average test results compared to the German standard population, with an average score of 11.42 (*SD* = 1.42) vs. 9.4 points (*SD* = 2.6) points (*p* <.001). Although students generally scored high, one question measuring the ability to visually “read beyond the data” yielded significantly lower scores and showed variability regarding the participants’ performance compared to other questions of this category.

**Conclusions:**

While abilities in visually “reading the data” and “reading between the data” are high in our cohorts, their ability to visually “read beyond the data” is inconsistent. This requires attention in the training of medical students, as weaknesses in this area could lead to susceptibility to misleading data. Enhancing graph literacy in medical students is crucial for effective physician-patient communication.

## Background

Information literacy depends on various skills in processing information [1]. A certain set of abilities is needed to understand medical reports, treatments, and study results adequately [2, 3, 4]. Thinking of future physicians, one can imagine a multitude of situations where high information literacy is required: talking with patients about medical data, consenting to treatments and educating patients about diseases, making clinical decisions depending on laboratory results, imaging, and study results, understanding evidence, interpreting epidemiological data, and communicating in medical teams [5, 6, 7].

A critical aspect of information literacy is visual graph literacy, which means reading, interpreting, and understanding graphs [8]. This process depends on decoding and interpreting signs and symbols, known as semiotic activity [9]. When information is provided graphically, the aim can be to make complex information more accessible and easier to grasp, but understanding it requires corresponding processing and interpretation abilities. Therefore, the ability to understand graphs should be considered in conjunction with other forms of information literacy. It is an integral part of processing and communicating information effectively in a world increasingly dependent on data and its visual representation.

Gaissmeier et al. [10] emphasize the significance of graph literacy in understanding health-related statistical information and the ability to make informed medical decisions. They found that high graph literacy is associated with better comprehension and recall of graphically provided information. For those with low graph literacy, numbers seemed to provide better comprehension and recall. This makes graph literacy tremendously important in patient-centered communication tools whenever patients show a possible lower graph literacy as healthcare professionals, as shown by Nayak et al. [11]. Processing those visual representations is essential for understanding scientific and statistical data [12]. This is particularly relevant in medical research, where graphs and data visualizations are frequently used to convey complex information. A personal understanding of the representations is fundamental when preparing data for communication to ensure adequate knowledge transfer to others [13]. But misleading representations (either through deliberate manipulation or unintentionally through errors or incompleteness) can also significantly influence the recipient’s reception of information [14].

In summary, graph literacy, as a form of semiotic activity, is a crucial component of overall literacy. It can impact risk comprehension [15], suggesting that higher graph literacy may be associated with better decision-making performance. A lack of understanding of visual representations can significantly impact decision-making for patients and (future) physicians. However, studies of graph literacy mainly refer to patients’ [16] or physicians’ ability [17] to interpret graphical representations. Data regarding undergraduate medical students’ abilities in graph literacy and investigations on how to increase these are lacking. To our knowledge, there is only one further study, that applied the graph literacy scale to undergraduate medical students in their final two years of medical school and to medical residents [18]. This study provides insights into graphical and numerical skills but focussed solely on advanced students in years 6 and above. Further, it took place at a private university, which might not be a transferrable setting for non-private universities in Germany. Our study aims to fill this gap by specifically examining undergraduate medical students in Germany.

Whenever healthcare professionals are tasked with informing patients, they must be aware of the patient’s ability to understand the provided information. Patients with stronger graph literacy can more sufficiently interpret information about their conditions, treatment options, and potential outcomes, leading to better-informed healthcare decisions [19]. However, there often exists a communication gap between healthcare providers and patients, where complex graphical data may not be presented in an accessible manner. Training that enhances undergraduate medical students’ graph literacy and their ability to convey this information effectively to patients is essential. By improving their skills in patient-centered communication, future physicians may empower patients to engage actively in their care, thereby improving adherence to treatment plans and, by this, improving overall medical outcomes [20]. To ensure adequate communication, they must also be able to assess their own ability to interpret the available information correctly. To gain a deeper understanding of undergraduate medical students’ ability to process graphically presented information, we conducted an observational cross-sectional survey study to understand their graph literacy. We aimed to investigate the following research questions:

1. What is the level of graph literacy among undergraduate medical students at a public German university?
2. Does the graph literacy of undergraduate medical students evolve during their academic careers at a public German university?
3. Are there specific areas of undergraduate medical students’ graph literacy that require particular focus at a public German university?

## Methods

### Study design and participants

We performed a cross-sectional study with undergraduate medical students in their academic years 1 to 5 at the medical school of Muenster University, Germany, to measure their graph literacy. It takes six years to complete a curriculum in medical school in Germany, with students enrolled directly from secondary schools. The curriculum of study is divided into a preclinical section (the first two years) and a clinical section (the last four years). To improve students’ clinical experience, they are rotated in various hospital departments during their final (sixth) year (“clinical/practical” year). We excluded year 6 students as they are engaged in practical rotations, which differ significantly from the academic focus of years 1–5. The study took place in rooms at the Muenster University during a mandatory exam. Students were asked to additionally answer the Graph Literacy scale pen and paper version before starting their written examination. Students were supervised while completing the Graph Literacy Scale. The written examination is limited to 4 h overall duration. The Graph Literacy Scale and the written examination had to be completed during this time frame.

Completion of the Graph Literacy Scale was voluntary and anonymous; calculators were not allowed. We did not ask participants about their social, biographical, or educational background. Still, a post-secondary school diploma is necessary for entering medical school, and a statistical curriculum is part of the education in German higher schools.

### Outcome measures

We measured the participants’ performance using the “Graph Literacy Scale” [21]. The students had to answer 9 numeric responses and 4 multiple-choice questions. Each question represents one of the 3 levels of visual graph comprehension: “reading the data” (4 numeric responses), “reading between the data” (3 numeric responses and 1 multiple choice question), and “reading beyond the data” (2 numeric responses and 3 multiple choice questions). The scale contains questions that, for example, ask the reader to analyze visual graphs regarding the efficacy of fictitious drugs in comparison (“reading between the data”) and reading off a point on a line chart (“reading the data”). An example of a numeric response is question 3 from the category “reading the data”: Respondents are presented a pie chart and asked to answer the question “Of all the people who die from cancer, approximately what percentage dies from lung cancer?” in numbers. The question aims to assess the ability to visually recognize a quarter of a pie and translate the information into a percentage. The next question (question 4) can be taken as an example for “reading between the data:” The respondents are asked to take information about three diseases from the same pie, thereby assessing their ability to sum up slices in a quarter of a pie presented visually. The category “reading beyond the data” asks respondents, for example, to project future trends from a line chart (question 7) or to compare two line charts attending to scale labels (question 11). Question 11 is accompanied by two graphics with line charts showing the outcomes of patients with psoriasis. The line charts lack sufficient axis labelling, making it appear as if one would show a better outcome than the other. The respondent is asked to answer the multiple-choice question “Which of the treatments contributes to a larger decrease in the percentage of sick patients?” Here, the correct answer would be “can’t say.”

Except for one question, which accepts a range of values (question 7), there is only one correct answer per question. Participants received one point for every correct solution, meaning they could achieve 0 to 13 points. Only the correct answer was considered, and only exactly defined numbers were considered correct in numeric response questions. There was no error margin, and incomplete questionnaires were not considered for our analysis. The average score in this test format, according to Galesic and Garcia-Retamero [21], is 9.4 (standard deviation (*SD*) = 2.6) for Germany. Their study included a representative sample of people 25 to 69 years of age in Germany from a panel of households contacted by a German company specialized in social research and statistical analyses. The German sample contained 74.1% people educated by high school or lower level of education and 25.0% educated by college or higher level of education. The gender ratios within the sample were balanced. Compared to pre-existing questionnaires, the test shows convergent validity for graph comprehension and correlates highly with numeracy skills. The graph literacy scale is internally consistent and reliable, with individual items and the total score correlating highly. We utilized the German version of the scale without modifications to the layout or the quality of the graphs.

### Statistical methods

We analyzed all data using the “R” programming language, version 4.4.0 [22] and calculated the mean test scores and standard deviations differentiated by sex, study year, and graph literacy dimensions. To analyze differences in test scores between the German general population and our samples, as well as between sub-samples, we performed Welch two-sample t-tests. Pairwise tests of equal proportions were conducted to investigate variability in the results of the sub-scale “reading beyond the data.” Bonferroni correction was applied to the results of the pairwise proportion tests to mitigate the risk of type I errors due to multiple testing. We performed multiple linear regression to analyze the effect of gender and study year on the outcome measure. The significance level was set to 0.05 for all statistical tests.

### Ethical approval

Approval was obtained from the local Ethics Committee University Muenster, Germany [2017-159-f-S], which examines the ethical admissibility of studies on the basis of the Declaration of Helsinki, which is enshrined in the German Medical Code of Conduct. By approving this study, the Ethics Committee University Muenster, Germany, confirmed that our study is in accordance with the Declaration of Helsinki. Every participant was asked for informed consent. A validated questionnaire for “Graph Literacy” was handed out to medical students from April 2017 to June 2017. Completion of the questionnaire was voluntary and took place after informed consent was given.

## Results

### Recruitment process and demographic characteristics

**Fig. 1:**
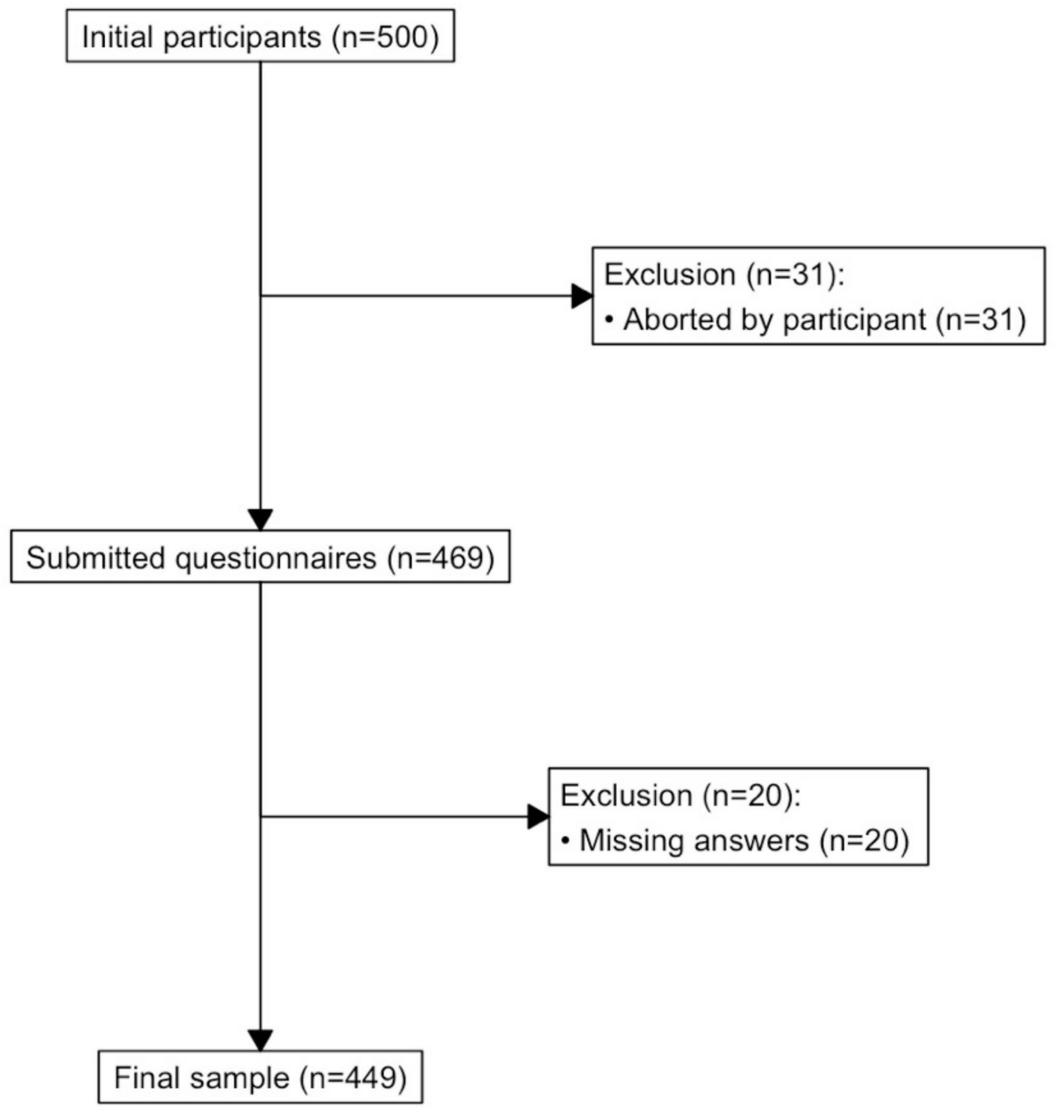
Flow chart regarding the inclusion of the available survey data

We obtained 449 complete questionnaires, which could be included in our analysis. Of a total of 500 Graph Literacy pen and paper scales started by students, 31 surveys were actively cancelled by the respondents. Of the 469 left, 20 lacked answers to individual responses and were excluded (Fig. 1).

Two questionnaires did not provide information on the sex of the respondents, and 1 did not provide information regarding age; those were included nonetheless. At the time of the study, there were approximately 1250 undergraduate medical students enrolled at Muenster University, distributed to 250 persons/study year (year 1 to 5). The gender distribution for the total population is approximately 65% female, 35% male.

Two students, in one case with 0 correct answers, in the second case with 3 correct answers, stand out. However, there were no indications of premature termination of the questionnaire or a lack of willingness to participate from the response behavior; thus, those questionnaires were considered valid responses. For participant details see Table 1. Gender distribution in our sample is corresponding to gender distribution of medical students at the University of Muenster. Due to data protection requirements, no exact data for age per participant is provided. Mean age and standard deviation can be found in the provided Table 1. In the original study for the validation of the Graph Literacy scale participants have been included from age 25 to 69. The age group 25–39 contained 31.4% of the German participants.

**Table 1:** Participant characteristics

Characteristic	*N*	Male, *N* = 151^*1*^	Female, *N* = 296^*1*^	Overall, *N* = 449^*1,2*^
Age^*3*^	448	22.8 (4.1)	22.3 (4.2)	22.5 (4.2)
Study year	449			
1		46 (30%)	80 (27%)	126 (28%)
2		35 (23%)	92 (31%)	128 (29%)
3		19 (13%)	35 (12%)	54 (12%)
4		19 (13%)	39 (13%)	59 (13%)
5		32 (21%)	50 (17%)	82 (18%)

^*1*^ Mean (*SD*); n (%)

^*2*^ Two participants (aged 19 and 23, study year 2 and 4) did not provide information regarding sex

^*3*^ One participant (male, study year 4) did not provide information regarding age

### Primary and secondary outcomes

**Fig. 2:**
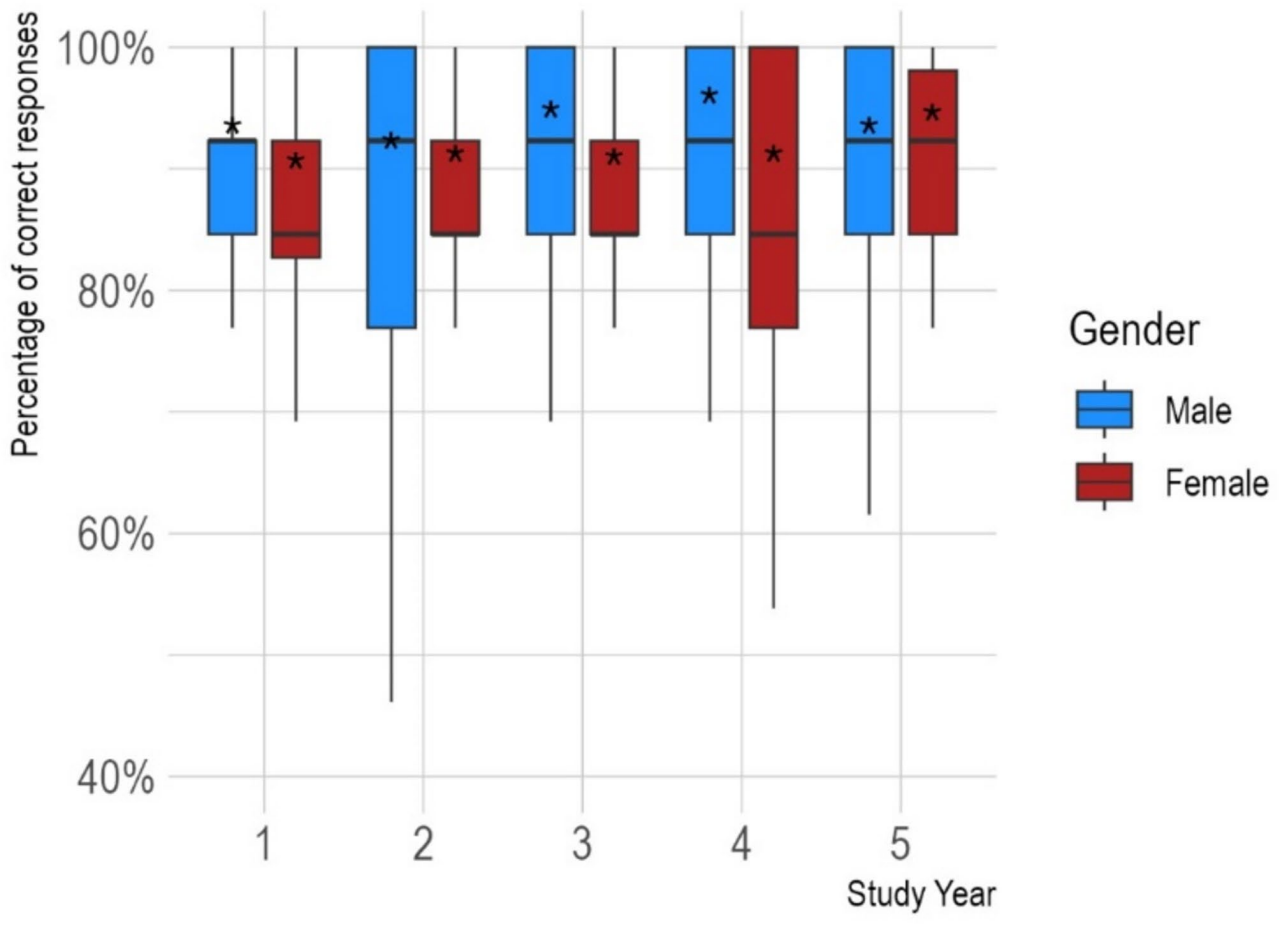
Boxplot of test performance by study year and gender. Horizontal bars denote the median value; the asterisks denote the mean

Participants achieved an average score of 11.42 (*SD* = 1.42) points, which is considerably higher than the average score reported by Galesic and Garcia-Retamero with 9.4 (*SD* = 2.6) points, *t*(790) = − 14.75, *p <.*001. Although we found a slight increase in scores between the first (11.33 points [*SD* = 1.40]) and the fifth academic year (11.65 [*SD* = 1.18]), the difference was not significant (*t*(192) = − 1.78, *p* =.076). Average test results by study year and gender are displayed in Fig. 2.

A bivariate comparison showed that male participants achieved significantly higher scores than female participants (male: 11.59 [*SD* = 1.32], female: 11.31 [*SD* = 1.51], *t*(341) = 2.01, *p* =.045). However, in multiple linear regression, gender (β = −0.27, 95% CI: −0.56 to 0.01, *p* =.061) was not a significant predictor of test performance after adjusting for study year (β = 0.08, 95% CI: −0.01 to 0.17, *p* =.10). While the model significantly outperformed the null-model (*p* =.041), it explained only a small proportion of the variance in test scores (adjusted R² = 0.01). This small proportion of variance in test scores, indicates limited practical significance and suggests that additional unmeasured factors may influence the outcome.

**Fig. 3:**
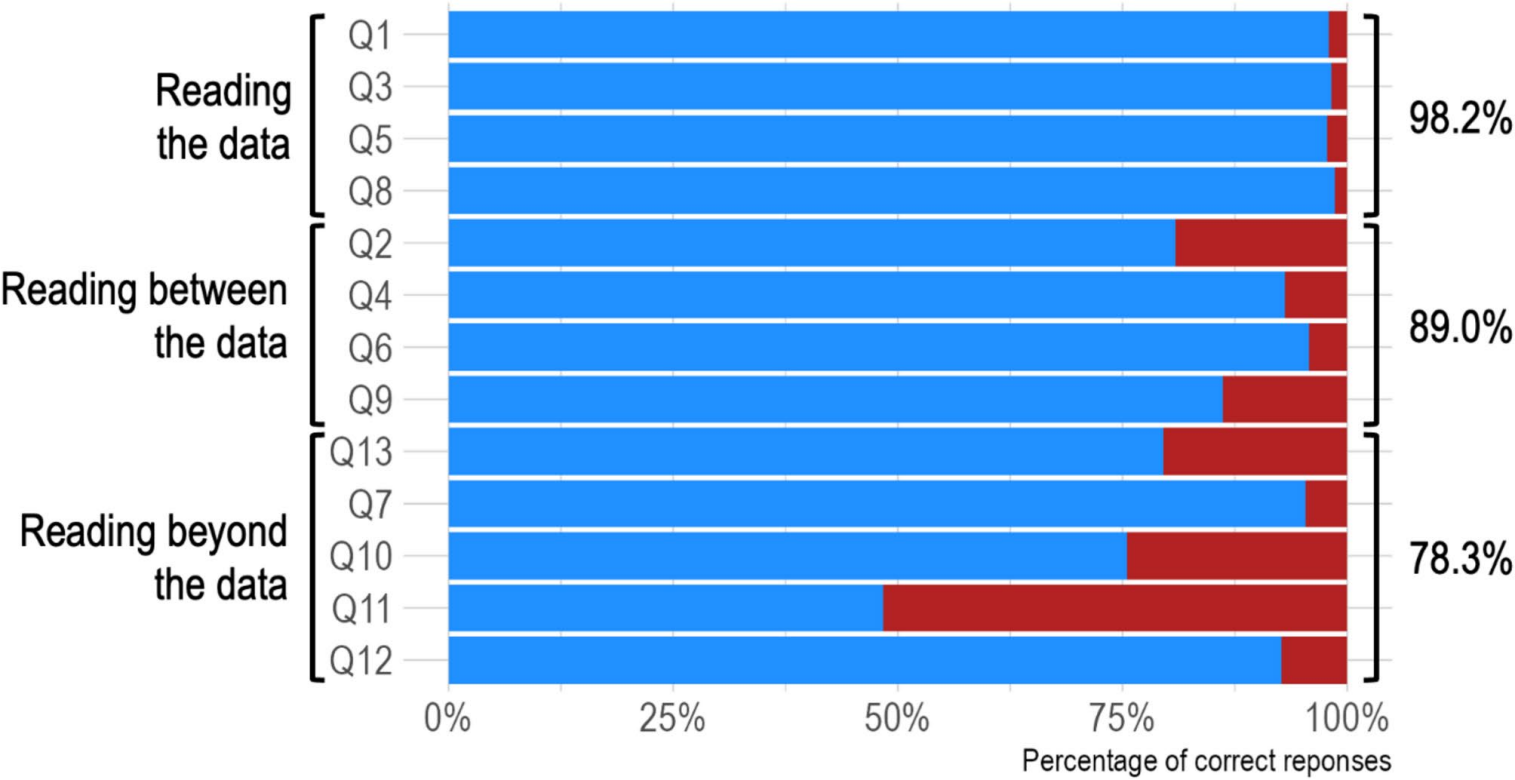
Mean percentage of correct answers for every Question (Q 1 to 13). Blue = correct answer, red = incorrect answer. The order of the questions corresponds to the evaluation order of the original Graph Literacy Scale. The categories (“reading the data”, “reading between the data”, “reading beyond the data”) to which the respective questions are assigned can be found on the left-hand side

**Fig. 4:**
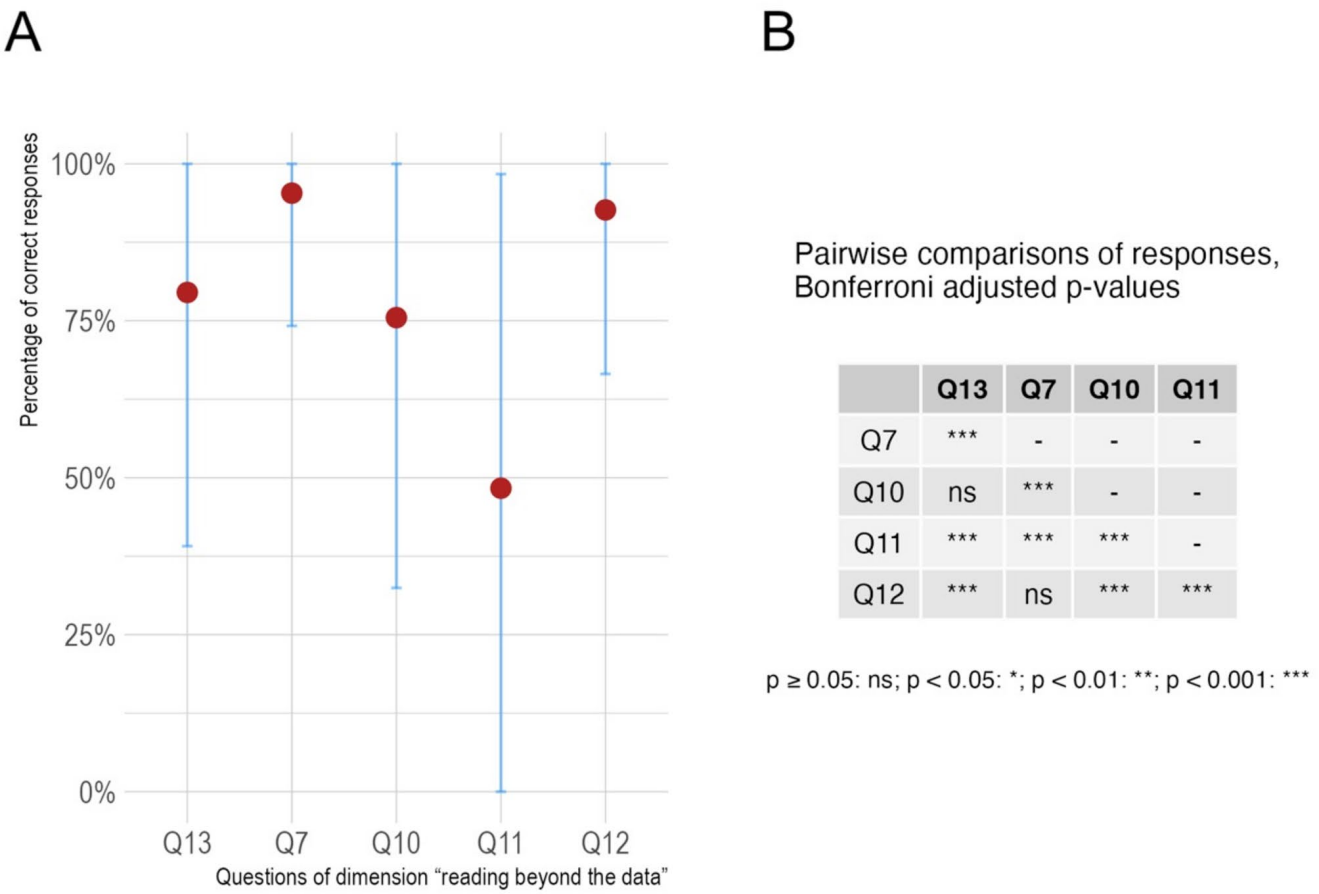
Performance variability regarding participants’ performance in questions belonging to the category visually “read beyond the data.” The proportions of correct answers per question and standard deviations are presented in panel A. Pairwise comparisons of proportions were performed, and p-values were subsequently adjusted using Bonferroni correction to mitigate the risk of committing type I errors. The adjusted significance of the differences of pairwise comparisons are presented in panel B

Finally, although the students in our sample achieved an overall mean percentage of 87.7% correct answers, question 11 yielded a percentage of 48.3% correct answers (Fig. 3). Question 11 tests the ability to visually “read beyond the data” and shows variability regarding the participants’ performance compared to other questions of this category (Fig. 4).

## Discussion

We used an established measurement instrument on a large sample of undergraduate medical students to determine their graph literacy. They exhibited above-average results on the “Graph Literacy Scale” compared to the German standard population. Our participants demonstrated a strong ability in “reading the data” with an average score of 98.2%, while their performance in “reading between the data” yielded an average score of 89.0%. This indicates a solid foundational understanding of graphical data representation. But while undergraduate medical students’ ability to read and interpret data is high, their ability to extrapolate visually provided information “beyond the data” is inconsistent, varying between individual questions, as demonstrated by the large variance between performance in questions belonging to the dimension “reading beyond the data.”

In graph interpretation, one can consider the three dimensions of the visual graph comprehension as translation (“reading the data”), interpretation (“reading between the data”) and extrapolation/interpolation (“reading beyond the data”). “Reading beyond the data” involves critical sense, analyzing data interrelations, and extrapolating information to answer implicitly presented answers to questions [23]. Therefore, despite outperforming the reference group, the lower performance in answering question 11 and the performance variance in the category “reading beyond the data” need further attention. In our study, undergraduate medical students in years 1 and 5 achieved 38.1% and 57.6% (average 48.3%) correct answers to question 11, respectively. This performance variability between the study years is consistent with research showing that especially visually “reading beyond the data” depends on users’ prior knowledge and skills in information comprehension regarding the graphically presented topic [24].

Question 11 requires comparing two line-charts to determine which treatment leads to a larger decrease in sick patients. The charts’ y-axes indicate “% sick patients” without providing a scale, making the correct answer “can’t say.” Due to our study design, the performance variance can only be described and not be conclusively explained. But it is important to be aware regarding this phenomenon: Intentional or unintentional misrepresentations of graphical information can be used to mislead physicians into misjudgements, e.g., convey certain medications as presumably more effective.

Two possible explanations for our observation emerge from other medical decision-making contexts. The observed high scores in other aspects of visually “reading beyond the data” suggest that performance on question 11 could hint at similar phenomena like a judgment and visual interpretation bias like a base-rate neglect or lack of ambiguity tolerance. Question 11 addresses, among other things, proportion judgment, without providing necessary information (a scale) for this. Proportion judgment is a known source of misjudgements in graph interpretation and a risk of visual bias [25]. A base-rate neglect is a cognitive bias where people tend to ignore or undervalue the base rate (general frequency) and instead focus on specific information related to the case at hand. For example, in cases of rare diseases, a physician might focus on the specific symptoms of the patient and neglect the low base rate (rarity) of the disease in the general population [26]. This can lead to overestimating the likelihood of the disease being present.

In the context of our study, base-rate neglect might manifest itself in undergraduate medical students finding it difficult to accurately interpret graphical data when the answer involves recognizing a lack of sufficient information to make a definitive conclusion, as seen in question 11. Combined with the lack of proportion information this could lead to incorrect answers due to ignoring the missing base rate and proportion information, indicating that the correct answer should be “can’t say.”

Whenever the correct answer is “can’t say,” a lack of tolerance for ambiguity could explain poor performance additionally. Graphic representations are intrinsically characterized by a certain ambiguity, as they can always cause user-dependent patterns of recognition and thus different interpretations despite the same perceptible representation [27]. Tolerance for ambiguity refers to the ability to accept uncertainty and cope with situations that lack clear answers. Research indicates a close association between low tolerance for ambiguity and perceived work-related stress in physicians [28]. Low tolerance for ambiguity can lead to premature decision-making, overconfidence in incorrect conclusions, or avoidance of complex problems [29]. Admitting uncertainty can be particularly challenging for physicians who must provide answers and guide therapy. Low tolerance for ambiguity is a known risk factor for suboptimal decision-making in clinical practice, as demonstrated by Saposnik et al. [30].

Undergraduate medical students with a low tolerance for ambiguity might struggle with a graphical representation where the correct answer is “can’t say” due to insufficient information. Their discomfort with uncertainty might lead them to choose a definitive but incorrect answer instead of acknowledging the ambiguity in the data. However, research on this topic is mixed. Eley et al. [31] found that ambiguity tolerance decreased during medical studies, suggesting students might perform worse on question 11 in later academic years, contrary to our findings. In contrast, Weissenstein et al. [32] reported no difference in ambiguity tolerance between first and sixth-year students in a German cohort. Additionally, Geller et al. [33] found that tolerance for ambiguity in undergraduate medical students changes over time, decreasing for those with initially high values and increasing for those with initially low values.

Ultimately, our findings remain inconclusive, and without directly trying to assess bias effects or measure tolerance for ambiguity in connection with graph literacy, any explanation remains speculative. Future studies should explore the role of cognitive biases, such as base-rate neglect and ambiguity intolerance, in graph literacy to provide empirical evidence for these speculations. Exploring the psychological factors influencing graph literacy in undergraduate medical students could be valuable for gaining a better understanding.

There are several further limitations to our study. First, we performed an observational cross-sectional survey study carried out in a limited time frame and the setting of a mandatory test every undergraduate medical student must take. This could have caused students to care less about the correct answers or to have a shorter attention span because they already had to exert much effort to perform well on their tests. Participants might have performed much better at a different time or later in their training, such as residents in clinical practice. There might be a training effect for graph literacy that only evolves over time, needing to read and explain data regularly to patients or colleagues in postgraduate medical education. Additionally, we did not ask participants to provide specific information regarding their statistical training in school or during their medical studies. The impact of more intensive training on the graph literacy skills in our cohorts remains unclear. While the difference between the first and fifth academic years was not significant in our sample, this aspect still deserves attention. Neither can we state conclusively why the performance regarding graph literacy was especially inconsistent in visually “reading beyond the data.” This question also requires further research.

However, an observational cross-sectional survey study design remains an efficient way to evaluate the prevalence of specific skills in a large sample of students. We cannot state how far graph literacy improves on the participant level as we only performed a singular measurement per participant. But we can say that, in comparison, those students with higher academic experience perform better than the German standard population when measuring their graph literacy and the differences between the analyzed study years may hint to a certain training effect on graph literacy with advanced university studies.

Few other studies provide insight into undergraduate medical students’ graphical and numerical skills. In a cross-sectional, descriptive study, researchers applied the “Objective Numeracy Scale,” “Subjective Numeracy Scale,” and “Graph Literacy Scale” to undergraduate medical students of a private university in their final two years of medical school and to medical residents [18]. The study included 169 participants, comprising 70% sixth-year and seventh-year students and 30% residents in Lima, Peru. The findings showed that the mean graph literacy was 10.35 (*SD* = 1.93) and thus significantly lower than in our study (*p* <.001). Multiple linear regression analysis in the Peruvian study revealed that higher scores on the Graph Literacy Scale were associated with male gender and younger age. The study concluded that the mean scores of the numeracy and graph literacy scales were high among the medical students in the sample, consistent with our findings of high graph literacy in our cohorts. Although our sample contains many more females than the validation study of the Graph Literacy scale used, we did not find significant gender differences. The regression model using gender and study year has little predictive power, as it explains only 1% of the variability in the outcome measure. This indicates that unaccounted variables affect test performance. Further sociodemographic data is needed for meaningful analysis in this regard.

Literature on patient and nurses’ graph literacy provides further insight with respect to the different levels of graph literacy. For example, Durand et al. [16] examined the relationship between graph literacy, numeracy, health literacy, and sociodemographic characteristics in a Medicaid-eligible population. They did not find a statistically significant association between graph literacy and higher education. However, they found a positive association between graph literacy and numeracy, while numeracy was associated with education. We did not ask students for information regarding their education. Still, due to the admission criteria for medicine studies in Germany, we do know that students at least have the highest possible university entrance qualification. Our study supports the assumption of a positive association between education and graph literacy in comparing our results with Galesic’s and Garcia-Retamero’s standard population data [21].

## Conclusions

Our findings suggest that while undergraduate medical students at a public German medical school generally exhibit above-average results in graph literacy, particularly in “reading” and “interpreting data,” they face challenges in visually “reading beyond the data” presented. Special attention should be given to improving their ability to correctly interpret visually presented information, as weaknesses in this area could lead to susceptibility to misleading data. Incorporating targeted graph literacy training into medical curricula could enhance students’ ability to interpret complex data effectively. Since physicians mediate between specialist knowledge and patient interests, enhancing this skill is crucial to ensure accurate and effective communication.

## Data Availability

The datasets used and analysed during the current study are available from the corresponding author on reasonable request.

## Abbreviations

CI: Confidence Interval
N: Number of participants
SD: Standard Deviation
